# Application of Probe-Capture metagenomics in rabies diagnosis

**DOI:** 10.1186/s12985-025-03029-6

**Published:** 2025-11-27

**Authors:** Panyan Liu, Zhenfeng Deng, Yingjin Wang, Fengwen Wu, JunSong Peng, Piwei Huang, Yuanli Wang, Jingmao Lao

**Affiliations:** 1 Infection Diagnosis Center, Guangxi KingMed Diagnostics, Nanning, China; 2 The First People’s Hospital of Qinzhou, Qinzhou, China

**Keywords:** Rabies virus, Probe-Capture metagenomics, Diagnosis, Rabies, Blood, Cerebrospinal fluid

## Abstract

**Background:**

Rabies, a lethal viral encephalitis caused by Rabies virus (RabV), is transmitted via bites, scratches, or mucosal contact with infected animals, as well as through inhalation of aerosolized particles, ingestion of contaminated raw animal products, or transplantation of infected organs. It’s near-universal fatality, diverse transmission routes, and marked clinical variability significantly impede timely diagnosis, highlighting the demand for a rapid and precise diagnostic approach.

**Methods:**

Single-center retrospective case series.

**Results:**

This series reported three rabies cases admitted to the First People’s Hospital of Qinzhou: one without identifiable exposure and two with confirmed exposure histories. Clinical presentations were highly variable and diagnostically misleading. Application of Probe-Capture Metagenomics (pc-mNGS) to cerebrospinal fluid and blood samples enabled direct identification of RabV and concurrent detection of coexisting pathogens.

**Conclusion:**

pc-mNGS demonstrates potential as a rapid, economical diagnostic tool capable of detecting RabV in specimens with low viral loads—such as blood and cerebrospinal fluid—from both exposed and unexposed individuals. Simultaneous identification of additional pathogens further supports its diagnostic utility.

**Supplementary Information:**

The online version contains supplementary material available at 10.1186/s12985-025-03029-6.

## Introduction

Rabies virus, a neurotropic single-stranded RNA pathogen, infects humans and other warm-blooded animals, causing rabies—a condition with an almost universally fatal outcome. Due to the development and extensive application of vaccines and immunoglobulin-based prophylaxis, the global incidence rate has significantly decreased [1–3]. In China, the incidence rate dropped from the highest rate of 0.250/100,000 in 2007 to the lowest rate of 0.009/100,000 in 2022 [4], and furthermore, the global strategic plan to end human deaths from dog-mediated rabies by 2030. Nevertheless, the World Health Organization reported in 2018 that rabies remains responsible for approximately 59,000 annual deaths, imposing considerable economic burdens [5]. Incidence rates are closely linked to socioeconomic status, with elevated prevalence in developing and underdeveloped regions [6]. Although definitive curative interventions are lacking, clinical observations from rare survivors and in vitro research suggest that early diagnosis, timely immunoglobulin administration, and prompt antiviral therapy may extend patient survival [7, 8].

Currently, diagnostic strategies for rabies primarily include virus isolation, antibody-based assays, immunochromatographic methods, and nucleic acid detection techniques. These conventional approaches typically necessitate a definitive exposure history and the manifestation of characteristic clinical symptoms, which may delay timely therapeutic intervention. Additionally, their diagnostic performance is often constrained by suboptimal sensitivity in specimens with low viral loads, such as blood and cerebrospinal fluid, limited feasibility in primary care contexts, and protracted turnaround times [9–11]. In intensive care settings, rabies virus-infected individuals frequently present with immunosuppression and heightened vulnerability to co-infections. Prompt identification and treatment of these secondary infections are imperative for patient survival. However, traditional rabies virus assays lack the capacity to detect concomitant pathogens, while routine diagnostic modalities for co-infecting agents, including smear microscopy and microbial culture, exhibit significant drawbacks—namely elevated false-negative rates and delayed results [12]. Hence, the implementation of a rapid, user-friendly, and broad-coverage diagnostic solution is critical for improving clinical management of rabies cases.

In recent years, the progressive refinement of sequencing technologies has substantially enhanced pathogen detection strategies. Among the leading approaches, metagenomic next-generation sequencing (mNGS) and probe-capture metagenomic sequencing (pc-mNGS) have demonstrated broad clinical applicability due to their unbiased detection capacity, scalability, rapid turnaround, and high accuracy [13–15]. The pc-mNGS method utilizes comprehensive probe libraries to minimize interference from abundant host-derived DNA in clinical specimens, thereby reducing the sequencing data requirements. Although the cost of mNGS has significantly decreased in recent years, it still presents a financial burden for patients. Currently, the cost of pc-mNGS is only half that of mNGS. A study by Sishi Cai et al. demonstrated that pc-mNGS exhibited superior sensitivity compared to mNGS in bloodstream infections (91.3% vs. 69.6%, *P* = 0.001). Additionally, research by Yuyao Yin showed that the performance of pc-mNGS in bronchoalveolar lavage fluid was comparable to that of mNGS. Our team’s latest findings revealed that in ocular infections, the overall detection rate of pc-mNGS exceeded that of mNGS, particularly for low-load pathogens, where it showed a notable advantage [12, 16, 17]. When considering parameters such as turnaround time, analytical sensitivity, and cost-effectiveness, pc-mNGS appears to outperform conventional mNGS in certain clinical settings, highlighting its significant potential for broader clinical adoption.

In this study, three rabies cases were identified via pc-mNGS, including one without a known exposure and two with confirmed exposure history. The findings demonstrate that pc-mNGS offers high sensitivity and precision in detecting rabies virus, independent of exposure status. Its broad applicability across diverse specimen types enables timely clinical diagnosis. Moreover, its capacity to identify co-existing pathogens supports clinicians in formulating comprehensive management strategies for rabies patients.

## Methods

In this retrospective case series, three patients who presented to the First People’s Hospital of Qinzhou between November 1, 2024, and February 1, 2025, were included. Informed consent was obtained from each patient and their families prior to participation.

Patients were stratified into two categories based on documented exposure history: those with a confirmed exposure and those without. Each subgroup was subsequently analyzed independently to account for potential etiological and clinical differences.

## Case presentation

### Unclear exposure history

#### Patient 1

**Fig. 1:**
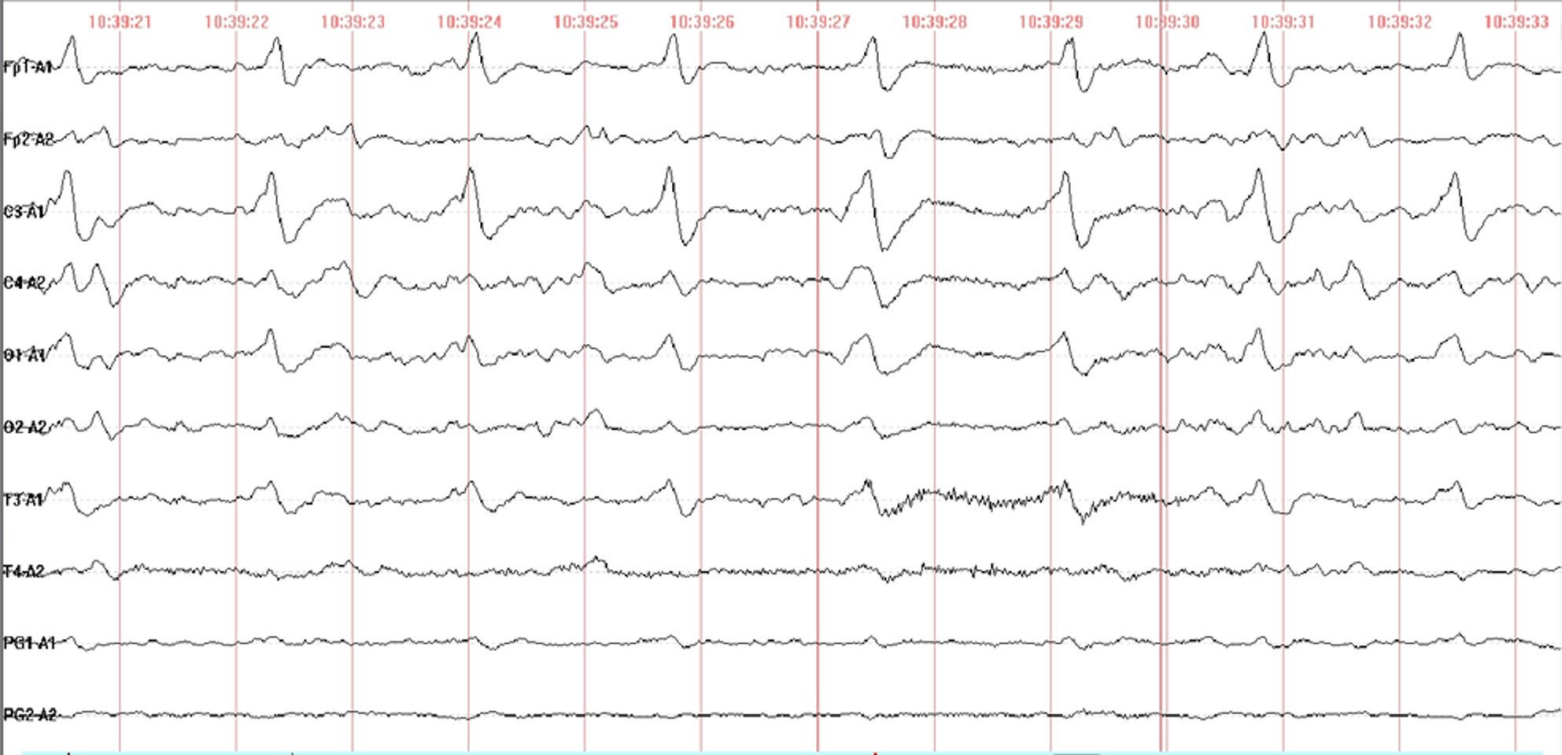
EEG profile of patient 1, marked by a background rhythm predominantly consisting of theta activity and widespread, severe rhythmic disorganization across all channels. The primary pathological discharges consist of recurrent, medium-amplitude sharp-and-slow wave complexes, exhibiting clear lateralization to the left hemisphere

**Fig. 2:**
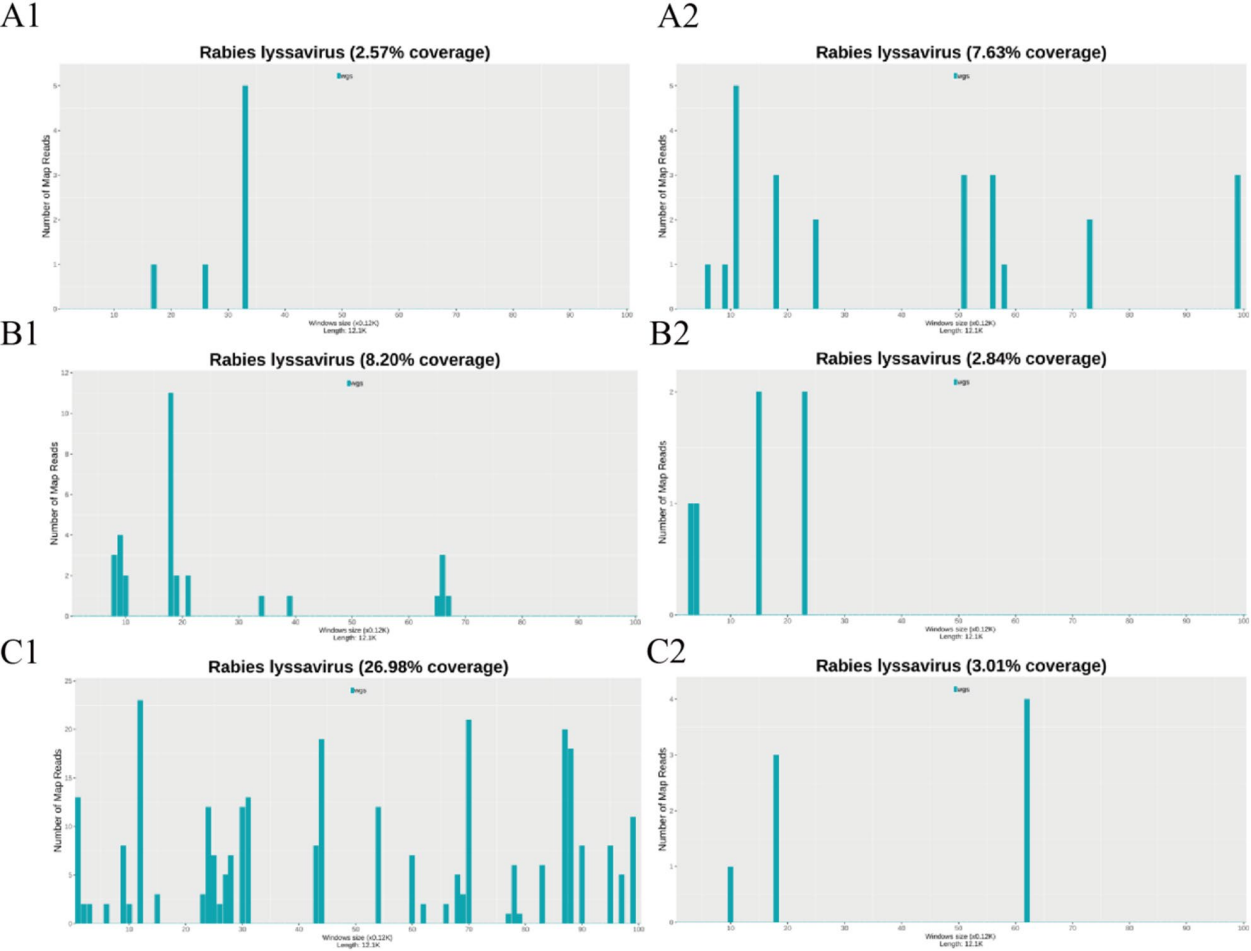
Genome Coverage of Rabies Virus Detected by Probe-Capture Metagenomic Sequencing. **A1**, CSF from Patient 1 (32 reads, 2.57%); **A2**, blood from Patient 1 (8 reads, 7.63%); **B1**, CSF from Patient 2 (27 reads, 8.20%); **B2**, blood from Patient 2 (6 reads, 2.84%); **C1**, CSF from Patient 3 (243 reads, 26.98%); **C2**, blood from Patient 3 (3 reads, 3.01%)

On November 10, 2024, a 56-year-old male patient presented with cough, sputum production, and fever peaking at 39 °C. Gastrointestinal symptoms, including abdominal pain, nausea, and gastric-content vomiting, subsequently emerged. As clinical deterioration progressed, he sought care at a local hospital, where empirical anti-infective and gastric mucosal protective therapies were administered. On the fourth day of admission, he experienced a single episode of coffee-ground emesis, followed by altered mental status and respiratory distress. Emergency airway management with endotracheal intubation and mechanical ventilation was initiated, after which he was transferred to this institution. Upon arrival, the patient was agitated, febrile (38.3 °C), tachycardic (140 bpm), and required vasopressor support (dopamine) to maintain blood pressure. Physical examination revealed mild conjunctival hyperemia and bilateral pulmonary moist rales. Laboratory evaluation demonstrated metabolic acidosis, hepatic and renal impairment, elevated myocardial enzymes, and coagulopathy. Cranial CT findings were unremarkable; however, chest CT indicated bilateral pneumonia. Sputum culture yielded *Klebsiella pneumoniae* (pneumoniae subtype). Given the suspected diagnosis of septic shock secondary to severe pneumonia, empirical antimicrobial therapy with meropenem and moxifloxacin was initiated. Despite treatment, inflammatory markers continued to escalate, and clinical status remained unimproved. Under sufficient sedation and analgesia, nociceptive stimuli failed to elicit any response, while intermittent muscular tremors gradually escalated into generalized seizures with associated nuchal rigidity. Lumbar puncture was undertaken to exclude central nervous system infections, yielding unremarkable cerebrospinal fluid (CSF) routine and biochemical profiles, with negative results for acid-fast and India ink staining. Electroencephalography (EEG) revealed marked abnormalities, typified by periodic lateralized sharp and slow wave complexes localized to the left hemisphere (Fig. 1). Persistent hypokalemia was noted, with electrocardiography (ECG) demonstrating diminished R-wave progression and QTc interval prolongation, consistent with electrolyte-related electrophysiological alterations. In pursuit of pathogen identification, blood and sputum specimens collected on November 19, 2024, were subjected to culture, and a blood sample was forwarded to Guangxi KingMed Diagnostics for pathogen capture metagenomic next-generation sequencing (pc-mNGS). On November 20, pc-mNGS reported *Acinetobacter baumannii* (64 reads), Rabies virus (8 reads, Fig. 2-A2), and Circovirus genus (3,725 reads). No history of animal bites or rabies vaccination was disclosed by the patient’s family. To substantiate the diagnostic inference, a second lumbar puncture was conducted on November 20, and the CSF specimen was analyzed via pc-mNGS, which subsequently detected 32 reads of rabies virus (Fig. 2-A1) on November 21. Concurrently, the sputum culture from November 19 confirmed multidrug-resistant *Acinetobacter baumannii*. Integrating clinical manifestations and sequencing data, a diagnosis of rabies was established. In response to the patient’s neurological and psychiatric status, valproic acid was administered to suppress seizure activity, and olanzapine was introduced for neuropsychiatric stabilization. On November 25, 2024, progressive hypotension and bradycardia ensued, culminating in death at 07:20.

### Exposure history

#### Patient 2

On September 6, 2024, a 55-year-old male presented with nausea, retching, and anorexia following acute alcohol intoxication and was evaluated at a local hospital. Despite initial intervention, neurological deterioration ensued, characterized by dysarthria and progressive hypertonia. By September 8, urinary and fecal incontinence emerged, accompanied by recurrent vomiting and febrile episodes, with emesis consisting of copious yellow-green bile. Owing to worsening clinical status, he was transferred to this facility on September 9 for further evaluation and management. On admission, the patient exhibited lethargy, pyrexia (38.2 °C), tachycardia (135 bpm), and hemodynamic instability necessitating dopamine infusion. Neurological examination revealed generalized muscle spasms and sustained hypertonia. The patient’s family reported a recent history of a dog bite without subsequent rabies immunization, in the context of chronic alcohol dependence. Comprehensive diagnostics identified pulmonary infection, respiratory acidosis, hepatic and renal impairment, and septic shock. On September 10, the patient experienced sudden cardiac arrest, coma, and diminished pupillary light reflex. Cardiopulmonary resuscitation, including endotracheal intubation and intravenous epinephrine, restored spontaneous circulation. On September 12, to investigate possible rabies virus infection and other coinfecting agents, blood and CSF specimens were submitted for high-throughput clinical metagenomic next-generation sequencing (pc-mNGS). The next day, pc-mNGS of the blood sample detected *Klebsiella pneumoniae* (8 reads), Epstein-Barr virus (EBV) (89 reads), cytomegalovirus (CMV) (22 reads), human herpesvirus 6B (HHV-6B) (8 reads), and rabies virus (6 reads, Fig. 2-B1), while the CSF sample revealed 27 reads of rabies virus (Fig. 2-B2). Despite ongoing therapeutic measures, the patient’s clinical course continued to decline. Following multidisciplinary discussions and in light of the prognosis, the family elected to withdraw life-sustaining treatment on September 15, 2024.

#### Patient 3

On January 17, 2025, a 53-year-old female patient presented with frequent urination, urgency, and dysuria and received oral norfloxacin at a community hospital. Symptomatic relief was minimal. By the early morning of January 19, 2025, she developed xerostomia, polydipsia, polyuria, dyspnea, palpitations, and limb weakness, prompting emergency admission. At presentation, she was alert, with a heart rate of 128 bpm and blood pressure of 166/89 mmHg. Electrocardiography indicated (1) sinus tachycardia, (2) left ventricular hypertrophy, (3) diminished R-wave progression, and (4) ST-T segment abnormalities. The clinical diagnosis included diabetes mellitus, hypertension, metabolic acidosis, respiratory alkalosis, and urinary tract infection. Glucose-lowering agents and antimicrobial therapy were initiated. Subsequently, progressive somnolence and respiratory decompensation developed, necessitating endotracheal intubation. Rising body temperature and elevated infection biomarkers led to the escalation of antibiotic therapy to meropenem. On January 21, 2025, the onset of intermittent limb tremors was noted, advancing to frequent seizures refractory to sedation and analgesia. Electroencephalography demonstrated severe abnormalities (refer to [Supplementary Figure]). The patient’s family reported domestic cat exposure, raising suspicion for rabies or tetanus. On January 23, 2025, blood and CSF samples were obtained for pc-mNGS to investigate potential causative pathogens. By the following day, pc-mNGS of the blood sample identified herpes simplex virus type 1 (HSV-1; 17 reads), rabies virus (3 reads, Fig. 2-C2), hepatitis B virus (2 reads), and background microorganisms such as circovirus (18 reads). The CSF sample yielded 242 reads of rabies virus (Fig. 2-C1). On January 25, 2025, the patient experienced cardiac arrest and cessation of spontaneous respiration, and death was declared at 8:45 AM.

## Discussion

Rabies is a lethal viral encephalitis transmitted predominantly via the saliva of infected animals. The incubation period typically spans 1–3 months, though it may vary from several days to multiple years. Initial symptoms often include fever, headache, anxiety, hydrophobia, and muscle spasms. As the condition advances, neurological deterioration may ensue, leading to altered consciousness, respiratory failure, and death. Despite substantial reductions in rabies incidence in many countries through extensive vaccination and immunoglobulin application, the disease persists as a significant public health burden in areas with limited vaccine accessibility [18–20]. Prompt diagnosis and early therapeutic intervention remain essential to improving patient survival.

This study presents three cases of advanced rabies infection, illustrating diagnostic complexity across distinct clinical scenarios. Case 1 involved a patient without identifiable exposure history who was ultimately confirmed to have rabies, reflecting the diagnostic difficulty in the absence of reported animal contact. Conversely, Cases 2 and 3, both with documented exposure to potentially infected animals, exhibited rapid symptom onset and progression, reinforcing the necessity of early suspicion and immediate clinical action.

Rabies presents with heterogeneous clinical features, progressing sequentially through incubation, prodromal, and acute neurological stages. Diagnostic confusion frequently arises due to symptom overlap with other neurological conditions, notably pseudorabies virus encephalitis. In the present analysis, initial clinical manifestations varied substantially among the three cases, complicating timely recognition. Case 1 developed respiratory complaints; Case 2 exhibited gastrointestinal disturbances; Case 3 initially showed signs consistent with urinary tract infection and diabetes-related pathology. This symptom diversity is consistent with prior reports indicating that early rabies presentations may include respiratory, gastrointestinal, and neurological disturbances, thereby increasing the risk of misdiagnosis and delaying definitive intervention [21–23]. The rapid clinical decline and emergence of septic shock further emphasize the necessity for early critical care support. Delayed pathogen identification and the absence of targeted therapeutic measures frequently result in unfavorable prognoses.

Current diagnostic modalities for rabies include virus isolation (via mouse inoculation or cell culture), antigen detection (e.g., fluorescent antibody test, direct rapid immunohistochemistry, ELISA), and nucleic acid detection methods (RT-PCR, loop-mediated isothermal amplification, and high-throughput sequencing). Virus isolation remains effective for specimens with high viral titers, such as brain tissue and saliva. Antigen detection techniques are primarily applied to brain tissue and nuchal skin biopsies. In contrast, nucleic acid detection methods offer greater versatility, enabling analysis of saliva, cerebrospinal fluid, blood, brain tissue, and skin biopsy samples.

In recent years, Next-Generation Sequencing (NGS) has emerged as a valuable technology for both pathogen identification and the investigation of various human diseases [24, 25]. mNGS, a key application of NGS, has demonstrated utility in detecting rabies virus not only in high-viral-load saliva specimens but also in CSF samples with comparatively lower viral concentrations, thereby contributing to early diagnosis and epidemiological tracing efforts for rabies [26–28]. A synthesis of relevant studies is presented in Table 1. Among sample types, saliva exhibits the highest mNGS positivity rate (85.71%), likely attributable to elevated viral titers that enhance detection sensitivity. In contrast, CSF samples display a delayed detection window and lower sequence yields, as observed in studies by Xu Lun Wang and Jing Wu [26, 28]. Consequently, in patients with confirmed exposure and clinical features consistent with rabies, saliva should be prioritized for NGS-based analysis. Nonetheless, saliva sampling presents limitations, particularly when evaluating the presence of non-rabies microbial species, as the clinical significance of such organisms remains difficult to interpret in this context. To date, mNGS-based detection of rabies virus in blood has not been reported, possibly due to the overwhelming presence of host DNA, which diminishes sensitivity for pathogen detection. This limitation is especially relevant for individuals with atypical presentations, including extended incubation periods or unclear exposure history, who may manifest systemic infection such as sepsis or septicemia during disease progression. Timely recognition of rabies remains challenging in clinical settings, particularly in the early stages when neurological manifestations are absent, often leading to prioritization of blood sample collection for pathogen screening. Neurological symptom onset typically prompts CSF testing; however, early detection of rabies virus in peripheral blood is essential for expediting diagnosis and initiating appropriate interventions. In this context, pc-mNGS provides comparable diagnostic capability to mNGS while mitigating several of its inherent limitations. By employing custom-designed, million-level specific probes capable of hybridizing with microbial nucleic acids, pc-mNGS enables efficient capture and enrichment of target genomic regions. The incorporation of both forward and reverse hybridization strategies further minimizes host DNA background, thereby enhancing detection sensitivity. In all three cases analyzed, rabies virus sequences were successfully identified in both CSF and blood specimens. Furthermore, we reviewed the previous reports on the use of standard mNGS for detecting rabies virus, and found that the rabies virus was not successfully detected in blood samples (Table 1).

**Table 1:** Summary of studies on the use of NGS to detect the rabies virus

	Gender	Age	Exposure history	CSF mNGS/hc-tNGS results (days)	Blood mNGS/hc-tNGS results (days)	Saliva mNGS/hc-tNGS results (days)	Survival period (days)
P1	Male	56	No	Positive(10)	Positive(9)	–	15
P2	Male	55	Yes	Positive(6)	Positive(6)	–	Unknown
P3	Female	53	Yes	Positive(5)	Positive(5)	–	8
P4	Female	49	No	Negative(unknown)	–	Positive(unknown)	10
P5	Male	49	Yes	Positive(7)	–	–	48
P6	Male	22	Unknown	–	–	Positive(4)	5
P7	Female	45	Unknown	Negative(1)	Negative(1)	Positive(15)	44
P8	Unknown	Unknown	Unknown	Positive(11)	–	–	Unknown
P9	Unknown	Unknown	Unknown	Negative(1,5,9), positive(23)	Negative(1,5,9,23)	Negative(1,23), positive(5,9)	29

Note: P, patient; P1–P3, Cases 1–3 in this text; P4, reference 26; P5, reference 27; P6–P9, reference 28; NA, not available; CSF, cerebrospinal fluid; Days, length of hospital stay

In addition to the aforementioned benefits, rabies patients exhibit a marked vulnerability to secondary infections. In all three reported cases, elevated inflammatory markers suggested concurrent infections. pc-mNGS identified *Acinetobacter baumannii* in the blood of Patient 1, with findings corroborated by sputum analysis collected on the same day; notably, pc-mNGS delivered results ahead of conventional sputum culture. Given the susceptibility of critically ill rabies patients to nosocomial infections in ICU settings, continuous surveillance of pathogen dynamics is essential. The cost of pc-mNGS ranges from one-fourth to one-half that of mNGS, offering a substantial reduction in financial burden and contributing to improved adherence to diagnostic protocols.

Preliminary evidence indicates that pc-mNGS holds promise as a more cost-efficient diagnostic modality for rabies, particularly effective in identifying low-abundance pathogens in blood and CSF, independent of documented exposure history. This approach also enables concurrent detection of additional pathogens and antibiotic resistance genes, thereby supporting clinical decision-making in rabies management. Nonetheless, due to the limited number of cases to date, further investigations are required to establish the method’s diagnostic specificity and sensitivity, and in future clinical work we will continue to monitor the consistency and discrepancies between pc-mNGS and conventional test results in rabies patients.

For long-term rabies prevention and control, expanded vaccination coverage and targeted public education are essential [29]. In parallel, large-scale epidemiological investigations are warranted to clarify transmission dynamics and optimize control measures. The present case highlights the diagnostic and therapeutic value of early recognition and timely medical intervention in suspected rabies infections.

## Supplementary Information

Below is the link to the electronic supplementary material.

Supplementary Material 1: Supplementary Table 1: Summary of the clinical data of the three patients

Supplementary Material 2: Supplementary Figure: EEG profile of patient

Supplementary Material 3: pc-mNGS protocol

## Data Availability

No datasets were generated or analysed during the current study.
