# Supplementary material for: Application of Probe-Capture metagenomics in rabies diagnosis
Source: Virol J. 2025 Nov 27;22:406. doi: 10.1186/s12985-025-03029-6 (PMC12752439; doi:10.1186/s12985-025-03029-6)
Supplement: Supplementary file 2 — Supplementary Material 2: Supplementary Figure: EEG profile of patient [file 12985_2025_3029_MOESM2_ESM.pdf]

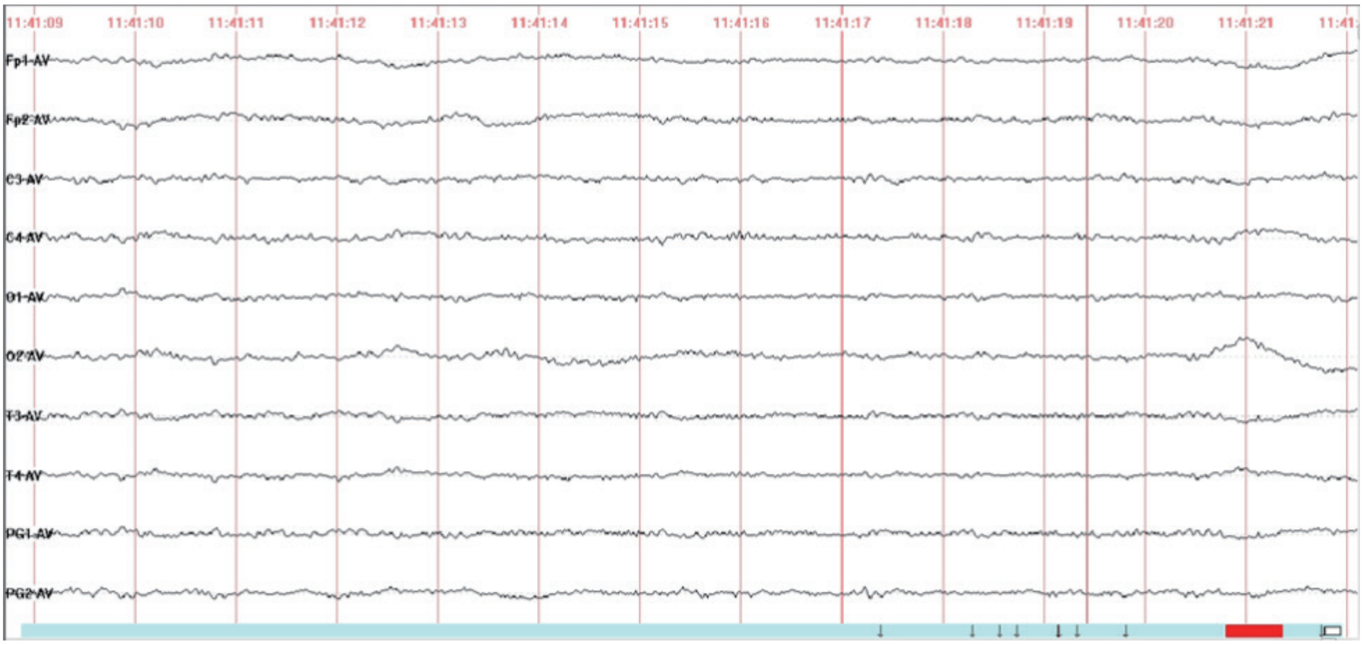

Supplementary Figure: EEG profile of patient 3 showing diffuse electroencephalographic abnormalities manifesting as increased β- and δ-band power.
