# Supplementary material for: Application of Probe-Capture metagenomics in rabies diagnosis
Source: Virol J. 2025 Nov 27;22:406. doi: 10.1186/s12985-025-03029-6 (PMC12752439; doi:10.1186/s12985-025-03029-6)
Supplement: Supplementary file 3 — Supplementary Material 3: pc-mNGS protocol [file 12985_2025_3029_MOESM3_ESM.docx]

**Methods**

①Sample collection and preparation

Blood samples (3 mL) were drawn from patients and stored at room temperature for 3–5 minutes before plasma separation, followed by centrifugation at 4,000 rpm for 10 minutes at 4°C. The plasma samples were then transferred to new sterile tubes. Cerebrospinal fluid (3 mL) was collected from the patients according to standard procedures. Plasma or cerebrospinal fluid samples were then immediately processed for the next steps.

②Nucleic acid extraction

The MagPure Pathogen DNA/RNA Extraction Kit (Magen Biotechnology, Guangzhou, China) was used to extract pathogen DNA and RNA from 300 μL of each sample, following the manufacturer’s instructions. Negative and positive controls were included in each batch to ensure the accuracy and reliability of the pc-mNGS workflow.

③Library construction and sequencing

We synthesized cDNA using a reverse transcription kit (KingCreate, Guangzhou, China) and prepared libraries with the corresponding library construction kit (KingCreate, Guangzhou, China). We pooled eight uniquely barcoded libraries and hybridized them with biotinylated probes from the MetaCAP™ Pathogen Capture Metagenomic Assay Kit (KingCreate, Guangzhou, China) for 30 minutes. We assessed library quality and concentration using the Qsep100 Bio-Fragment Analyzer (Bioptic, Taiwan, China) and the Qubit 4.0 fluorometer (Thermo Fisher Scientific, MA, USA), respectively. We then diluted and denatured the libraries and sequenced them on the KM MiniseqDx-CN platform using a universal sequencing reagent kit (KS107-CXR, KingCreate, Guangzhou, China). Each library generated approximately 1 million single-end reads, with a read length of 100 bp.

④Bioinformatic analysis

We processed the raw sequencing data using Fastp (version 0.23.1) to remove adapters, low-quality reads, sequences containing more than 5 ambiguous bases, and reads shorter than 35 bp, thereby obtaining high-quality clean reads [1]. We then aligned the clean reads to the human reference genome (hg38) using BWA (Burrows–Wheeler Aligner, version 0.7.17r1188) and removed human-derived sequences[2]. The remaining reads were compared against a microbial reference database, which included 11,958 bacteria, 4,414 RNA viruses, 2,959 DNA viruses, 1,714 fungi, and 343 parasites. Finally, we calculated species- and genus-level abundances based on reads per million sequencing reads (RPM).

⑤Interpretation of pc-mNGS results

We evaluated the performance of pc-mNGS based on normalized reads, the negative control ratio (NCR), and clinical relevance. Normalized reads were defined as the number of specific microbial sequences per million sequencing reads (RPM). To prevent contamination, we considered a result to be true positive when the NCR of a species or genus was ≥10. We defined NCR as the RPM of a given microbial sequence in the sample being at least tenfold higher than that of the same sequence in the negative control.

**References**

1. Chen S, Zhou Y, Chen Y, Gu J: **fastp: an ultra-fast all-in-one FASTQ preprocessor.** *Bioinformatics* 2018, **34:**i884-i890.

2. Li H, Durbin R: **Fast and accurate short read alignment with Burrows-Wheeler transform.** *Bioinformatics* 2009, **25:**1754-1760.
